# Comparative response of some tropical maize hybrid and their parental varieties to low and high nitrogen regime

**DOI:** 10.1016/j.heliyon.2021.e07909

**Published:** 2021-09-02

**Authors:** Sunday Ayodele Ige, Omolaran Bello, Stephen Abolusoro, Charity Aremu

**Affiliations:** a Crop and Soil Department Landmark University, Omuaran, Kwara State, Nigeria; b Department of Agronomy, Federal University Gashua, Nigeria

**Keywords:** Hybrid, Varieties, Open pollinated, Low and high-N

## Abstract

The study was carried to investigate the differential response of some maize hybrids and their open pollinated parents to low and high nitrogen soil condition. Ten open-pollinated varieties were crossed in a partial diallel fashion to generate 45 F_1_ hybrids during the 2011 cropping season at the International Institute of Tropical Agriculture (IITA), Ibadan, Nigeria. The 45 F_1_ hybrids and the ten parents were evaluated in four environments at Mokwa and Zaria in August, 2012 under high and low N conditions with two different levels of nitrogen application (30kgha^-1^ and 90kgha^-1^) respectively.

Hybrids recorded shorter plant height under the two different nitrogen fertilizer conditions compare with open pollinated parents. Thus the hybrids are better for mechanical harvesting. Taller ear height was observed among the hybrids compare with the open pollinated parents; rate of stem and root lodging were consequently higher among the hybrids. Hybrid had better husk cover under both nitrogen fertilizer regime; that's they will be less susceptible to insect and animal attack.

Mean difference of ear per plant between the maize hybrids and open pollinated parents was not significant. Significant difference was observed between maize hybrids and their parents for grain yield under low and high fertilizer regimes. That the hybrids had higher grain yield under both nitrogen fertilizer regime; indicates the tolerance of maize hybrid to low-N condition than the parental varieties. Low-N environment seemed to favor shorter day to silk for maize hybrid, however, high nitrogen soil condition appeared to increase day to silk. It is an evidence that flowering and consequently maturity time is delayed under favorable nitrogen soil environments.

Days to 50% pollen shed are significantly different among the maize hybrids and their parents. The maize hybrids attained day to pollen earlier than the parents under both fertilizer regime. It is an indication that setting of maize grain occur earlier among hybrids than their open pollinated parents.

## 1 Introduction

Tropical soil is mostly identified with low nitrogen level as result of continuous cropping which eventually resulted to low crop yield [1]. Nitrogen is very important for maize production including all the grass family. Nitrogen is vital in the utilization of other two principal plant nutrient i.e. phosphorus and potassium [2]. Nitrogen is easily lost from the soil through leaching and volatilization. Nitrogen fertilizer are not easily available to some of the local farmers due to unavailability, cost of procurement, and lack of technical know how about its application [3].

Development of low-N tolerance varieties is therefore considered as the best alternative way to improve maize grain yield in low nitrogen environment in the tropics and consequently reduce the residual effects of synthetic nitrogen fertilizer application on animal and human being.

Maize genotypes response differential to nitrogen application in terms of absorption and utilization [4, 5, 6, 7, 8, 9]. [10] in an experiment conducted to compare the performance of maize hybrid and the open pollinated one, the author concluded that hybrids gave higher grain yield, and that utilization efficiency of nitrogen is higher in hybrid than open pollinated variety. Banjoko and Adediran [11] similarly conducted a study to determine, the effect of source, rate and method of nitrogen fertilizer application on maize yield in the savanna zone of south western Nigeria. The author observed that there were no significant differences in yields obtained from the fertilizers applied. Also, the methods of application did not influence the yield of maize. Significantly. He however observed that the rates of nitrogen fertilizer applied significantly influence the maize grain yields. He therefore concluded that the application of 100 kg of nitrogen per hectare produced higher maize grain yield, and that of nitrogen content in the ear and leaf tissue. The author also observed that low soil nitrogen significantly decrease maize grain yield, among older maize varieties.

Matusso, J. M [12], conducted an experiment to investigate the effects of different levels of nitrogen fertilizer on the growth and yield of maize. The study revealed that maize growth parameters such as plant height, ear height, stem girth, and ear length increased significantly with increase in nitrogen application level [13]. conducted a study to evaluate the performances of five open pollinated maize varieties tested under two different levels of nitrogen fertilizer application (0 and 100 kg N/ha). The study revealed that nitrogen fertilizer application significantly increased the growth, maize grain and related traits except, ear height and ear length.

Another author [14] carried out a study to investigate the influence of nitrogen, boron and zinc as basal and foliar application on growth and yield of maize. The experiment comprises two levels of Nitrogen (120 and 150 kg ha-1), Boron (5 kg ha-1) and Zinc (25 kg ha-1). The author observed higher plant height, dry matter weight, crop growth rate, number of cobs per plant, and number of grains per cob, at 150, 75 and 60 kg/ha of N, P and K respectively for tested maize varieties. Studies on the comparative response of hybrid maize and their parents to different nitrogen levels are still very scarce in journals.

This study was therefore carried out to investigate the differential response of maize hybrids and their open pollinated parent to low and high nitrogen environments.

## 2 Materials and methods

Ten open-pollinated varieties developed at two different breeding eras (1979–1999, 2000–2008) regarded as era 1 and 2 were crossed in a partial diallel fashion to generate 45 F_1_ hybrids during the 2011 cropping season at the International Institute of Tropical Agriculture (IITA), Ibadan, Nigeria. The resultant hybrids were harvested, processed, fumigated and stored in the cold room prior to field evaluation. The 45 F_1_ hybrids and the ten parents were evaluated in four environments at Mokwa and Zaria in August, 2012. Mokwa (Latitude 9° 18^1^N, Longitude 5^0^N 4^1^E) and Zaria (Latitude 12^0^00^1^N, Longitude 8° 22^1^E) (Guinea savanna) with friable, deep sandy clay loam texture. where the genetic materials were evaluated under high and low N conditions with two different levels of nitrogen application (30kgha^-1^ and 90kgha^-1^) respectively. . Also, the textural characteristics, bulk density and porosity in the this locations where the studied were carried out showed good structured development in the sub-horizon (20–40 cm), which favour good agricultural production.In all evaluations, two row plots were used. Each row was 6m in length, spaced at 0.75m between rows 0.25m within rows with four replications to give a population density of approximately 53,333 plants per hectare. Data were collected on grain yield (t/ha), days to 50% pollen shed, days to 50% silking, anthesis silking interval, plant and ear heights, root and stem lodging (rated 1–9), husk cover (rated 1–9), plant and ear aspects (rated 1–9), ear harvest and number of ear per plant.

All measured data were subjected to combined analysis of variance (ANOVA). Means were separate by the use of least significant differences (LSD) according to Steel & Torrie (1980).

## 3 Results

The two different nitrogen levels were significantly different for grain yield, days to 50% anthesis and 50% silking, root and stalk lodging, ear aspect and number of ears per plant (Table 1). Variation among the ten open pollinated varieties was significant for days to 50% anthesis and plant and ear heights under low-N and high-N environments. Hybrids were significant different from each other for grain yield, anthesis-silking interval, plant height and husk cover. Parent versus hybrid effects were significant for grain yield, days to 50% anthesis, stalk lodging, plant and ear aspects. Genotypes x nitrogen levels effect were not significant for any traits measured under low-N and high-N environments.

**Table 1:** Analysis of variance for grain yield and agronomy traits in F_1_hybrids and Parents.

Sources	DF	Yield (t/ha)	Anthesis (Days)	Days to Silk (Days)	Anthesis silking interval (Days)	Plant Height (cm)	Root lodging (%)	Stalk lodging (%)	Husk cover (1–9)	Plant aspect (1–9)	Ear Harvest (no)	Ear Aspect (1–9)	Ear per plant (no)
N/level	1	2.95∗	203.84∗	69.05∗	6.06	4387.76	1.36∗∗	3.14∗∗	2.53	0.00	180.61	11.12∗	0.06∗
Rep/N	6	7.54	12.97	92.96	3.05	34184.20	0.16	0.01	3.04	16.64	434.97	13.20	0.22
Genotype	54	1.52	35.41	352.98	1.19	153900.00	0.01	0.01	0.73	0.79	177.27	1.26	0.15
Parent(P)	9	0.73	18.97∗	203.73	0.56	30543.4∗	0.01	0.01	0.73	0.33	1355.2	1.07	0.69
Cross(C)	44	0.81∗	35.53	139.15	0.61∗∗	532.42∗	0.01	0.01	0.68∗	0.70	41.29	1.35	0.03
P vs C	1	36.53∗∗	339.72∗∗	24.87	5.83	2043.95	0.01	0.06∗	2.28	4.91∗	964.50∗	9.22∗	0.99∗
Gen x N	54	0.44	9.27	75.59	0.01	3361.18	0.01	0.01	0.38	0.64	137.80	1.00	0.17
Error	324	1.88	32.55	87.76	0.65	4018.00	0.03	0.01	0.86	0.92	204.70	1.21	0.20

∗,∗∗ Significant at < 0.05 and 0.01 levels of probability respectively.

Means and ranges in the means for grain yield and agronomic traits across Low-N environments (Mokwa and Zaria) are presented in (Table 2). Higher grain yields were recorded among the hybrids compared to the parents under low-N condition, which was similar to grain yield recorded for each group in stress-free environments. The hybrids flowered earlier than parental populations by two (2) days but the parents produced higher ears per plant than the hybrids. There were no differences in the ratings for husk cover, plant and ear aspects for both hybrids and parents. However, root and stalk lodging incidences were more severe among the hybrids compared to the parents. Means and ranges in the means for grain yield and agronomic traits under high-N environment were similar to those obtained under the Low-N and stress free environments, with higher mean grain yield obtained in the F_1_ hybrids relative to the parents (Table 3). Grain yield ranged from 1.6 t/ha^−1^ to 2.98 t/ha^−1^ for hybrids and 1.2 t/ha^−1^ to 2.2 t/ha^−1^ for parents in high-N environments. In contrast to results obtained for the low-N environments, days to flowering appeared to be earlier among the parental populations compared to the hybrids under high N environments. The range in the means for plant and ear heights under high-N environments, were 39cm and 23cm respectively for hybrids compared to 106 cm and 41cm in parents. Ranges in the means for husk cover, plant aspect and ear aspect ratings were similar for both hybrids and parents respectively across high-N environment, which was similar to results obtained under Low-N environments. ASI for hybrids and parents was (2 days) and the values ranged between 1 and 2 days for both hybrids and parents. There were also no differences in the ratings for root and stalk lodging incidences between hybrids and parents in these environments.

**Table 2:** Means and ranges in the means for grain yield and agronomic traits in the tested genotypes across Low-N environment (Mokwa and Zaria).

Trait	Mean		Range	
	Hybrids	Parents	Hybrids	Parents
Days to anthesis	64.1	65.5	0.6–0.9	0.6–1.7
Days to silk	66.2	67.4	62–69	64–69
Plant height	159.0	159.3	145–177	146–200
Ear height	71.3	68.3	61–90	59–76
Husk cover	2.9	3.0	2–3	2.5–3
Plant aspect	4.1	4.2	4–5	4–5
Ear harvest	25.4	21.1	22–30	18–27
Ear aspect	3.8	4.0	2–3	3.4
Athesis-silking interval	2.0	2.0	1–3	1–3
Root lodging	0.2	0.1	0.01–0.02	0.1–0.2
Stalk lodging	0.1	0.1	0.03–0.2	0.06–0.1
Stagy green 10WAP	4.2	4.1	4–5	4–5
Ear number per plant	0.7	0.8	57–67	62–67
Grain yield (t/ha^−1^)	2.1	1.6	2.-3	1–2

**Table 3:** Means and ranges in the means for grain yield and agronomic traits in the tested genotypes across high-N environment (Mokwa and Zaria, 2012).

Trait	Means		Range	
	Hybrids	Parents	Hybrids	Parents
Days to anthesis	64.63	63.9	56–58	57–69
Days to silk	66. 0	66.9	52–69	64–69
Plant height	162.2	169.8	140–179	137–243
Ear height	72.1	70.6	59–82	57–98
Ear harvest	23.9	22.8	20–29	14–28
Ear aspect	3.9	4.3	3–5	4–5
Anthesis silking interval	1.8	1.6	1–2	1–2
Root lodging	0.3	0.2	0.1–0.3	0.2–0.3
Stalk lodging	0.2	0.2	0.2–0.3	0.1–0.3
Ear per plant	0.7	0.8	0.6–0.9	0.7–2
Grain Yield (t/ha^−1^)	2.3	1.7	1.6–3.0	1–2

## 4 Discussion

Majority of tropical soil is highly deficiency of nitrogen which happen to be one of the principal nutrient required for plant growth. This actually resulted from continuous cropping that eventually degraded the soil of nitrogen and other vital nutrient that are needed for better crop yield [15]. And moreover, because of the environmental hazard that result from continuous application of synthetic nitrogen fertilizer to improve soil nitrogen to require status, development of low-N tolerant varieties has been best alternative to boost crop yield in the tropics. Highly significant differences among the genotypes tested in this study for grain yield, anthesis, and days to silk, husk cover, number of harvestable ears per plot and ears per plant under low-N environments, indicate the presence of sufficient genetic variability among the populations sampled. This indicates the availability of sufficient genes that can be selected or manipulated for the hybridization for these traits, most especially for grain yield under low-N conditions. The components of the mean squares for all the traits under low-N condition also differed significantly, a situation which was similar to observations recorded under high-N environment for all the traits except for days to 50% silking, root lodging and ASI. [16], in their own study of genetic variability for nitrogen use efficiency in the research farms of the Nigerian Institute for Oil Palm Research (NIFOR) in the south western part of Nigeria, also observed differences in the expression of the genotypes under low and high-N condition. They therefore concluded that the different environments exerted influence on the expression of the especially (grain yield and ear per plant). However, that the components of G x E interaction mean squares for all agronomic traits were not significant across low and high-N environments, indicated that the 10 genotypes responded similarly to the two different levels of N-fertilizer application at the two locations (Zaria and Mokwa).

The expression of maize plant height under low and high-N conditions also showed that genotypes within Era 2 were shorter under low-N than under high-N conditions indicating differential response to N-availability by genotypes within this group. This report agrees with that of [17] who noted that certain morphological and physiological responses of maize under N deficiency conditions are usually expresses through reduction in plant height, low efficiency in light interception, accelerated senescence, increment in N mobilization to grain and reduction in N concentration in plant. Yield reduction under low-N was also observed among the genotypes in era 2 compared to their performance under high-N T, which is in line with earlier studies of [18] and [19] who reported that one of the main effects of N stress is a reduction in the photosynthesis structures components levels, e.g. chlorophyll, resulting in a reduction in the photosynthesis capacity also in carboxilase efficiency and, therefore low yield.

Genotype x environment (GE) mean square effects for grain yield and most of the agronomic traits were greater in magnitude under high-N than in low-N. The high GE variance component for grain yield and agronomic traits in high-N environments emphasized the need for multi-environment testing in order to identify nitrogen-use efficient genotypes with broad adaptation to different levels of N availability. Several studies [16, 20, 21, 22], have shown that the superiority of a cultivar in a particular environment may not be repeatable in another environment as a result of the response of a genotype to factors peculiar to specific environment. Consequently, an additional effect apart from genetic and environmental effects resulting from their interaction (i.e. G x E) is then detected. Within era 1 mean squares which were only significant for days to 50% anthesis and silking across the N levels also suggests that environmental factors did not influence the expression of most of the traits within each era. Furthermore, that Era 1 versus era 2 and (Era 1 versus era 2) x environment mean square components were not significant for all the traits measured under low and high-N, suggests that there were no substantial differences between the means of the genotypes in the two eras and that their performances remained stable across the two locations.

ASI is a useful indicator in screening genotypes for tolerance to stress because it is a measure of nicking (synchronization) of pollen shed with silking. Genotypes within era 2 had shorter ASI with corresponding higher grain yield across low and high-N environments compared to genotypes within era 1. In an earlier study in Canada [23], reported that most significant changes in the new Ontario, Canada hybrids was a tendency for a higher final leaf number, a longer duration from planting to tassel emergence and reduction in ASI.

Highest grain yields 1.87t/ha were observed among era 2 compared to 1.79t/ha in era 1 genotypes across low-N levels which was accompanied by earliness (i.e. shorter days to silking) and lower root lodging. In their own study [24], observed that genotypes that had shorter days to silking had longer grain filling periods, a phenomenon which may have contributed to increase in grain yield of era 1 genotypes. Similar observation was also made across high-N environments with grain yield of (2.15t/ha) in era 2 compared to (1.88t/ha) in era 1.

**Figure 1:**
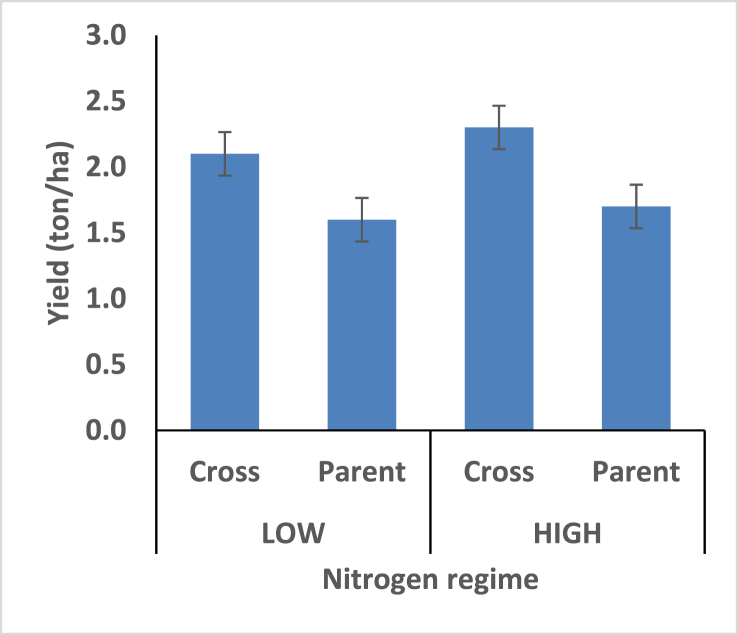
Effect of interaction between nitrogen fertilization and maize hybrids, open pollinated variety on grain yield.

**Figure 2:**
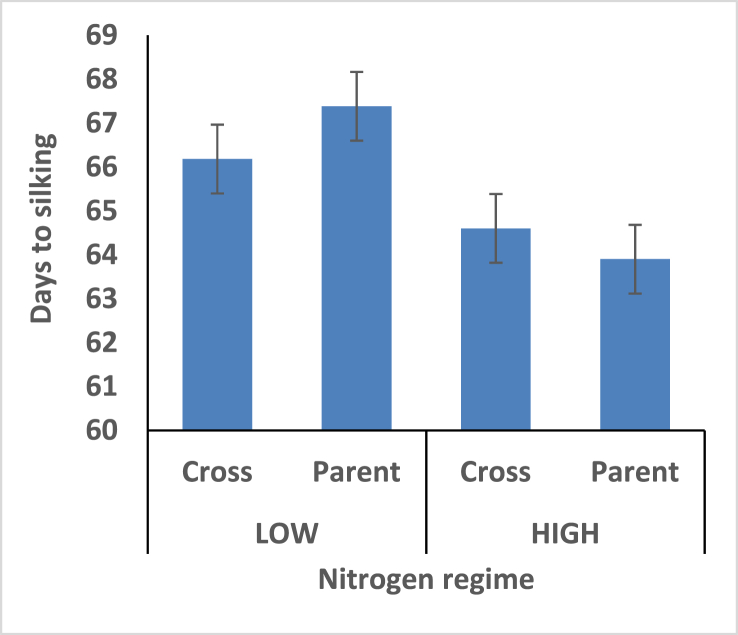
Effect of interaction between nitrogen fertilization and maize hybrids, open pollinated variety on day to silk.

Figure 1: revealed a significant difference between maize hybrids and their parents for grain yield under low and high fertilizer regimes. The hybrids had higher grain yield under both nitrogen fertilizer regime. Indicates the tolerance of maize hybrid to low-N condition compare to parental varieties. Low-N environment seemed to favor shorter day to silk for maize hybrid, however, high nitrogen soil condition appeared to increase day to silk (Figure 2). It is an evidence that flowering and consequently maturity time is delayed under favourable nitrogen soil environments.

**Figure 3:**
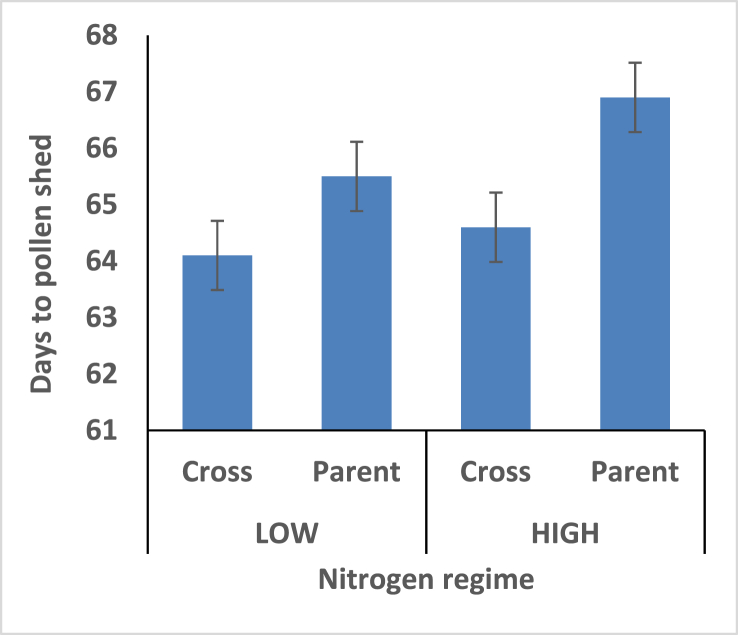
Effect of interaction between nitrogen fertilization and maize hybrids, open pollinated variety on pollen shed.

Days to 50% pollen shed are significantly different among the maize hybrids and their parents (Figure 3). The maize hybrids attained day to pollen earlier than the parents under both fertilizer regime.

**Figure 4:**
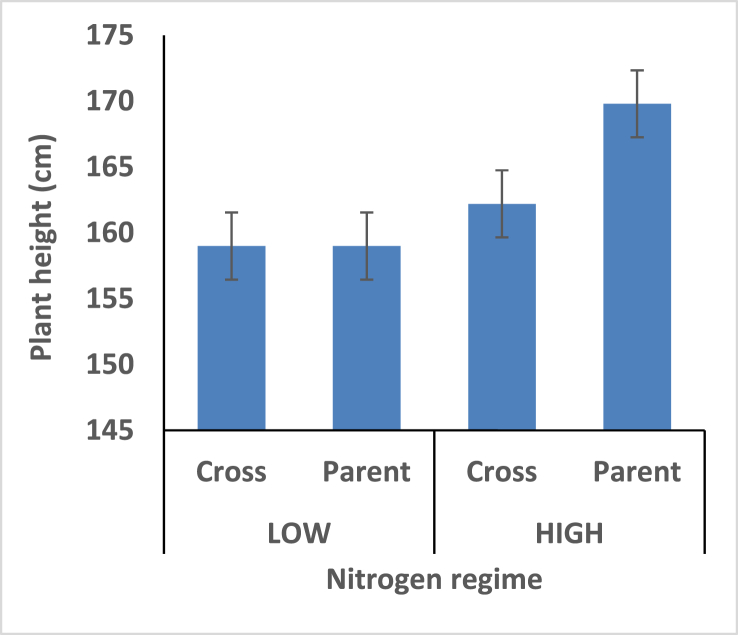
Effect of interaction between nitrogen fertilization and maize hybrids, open pollinated variety on plant height.

**Figure 5:**
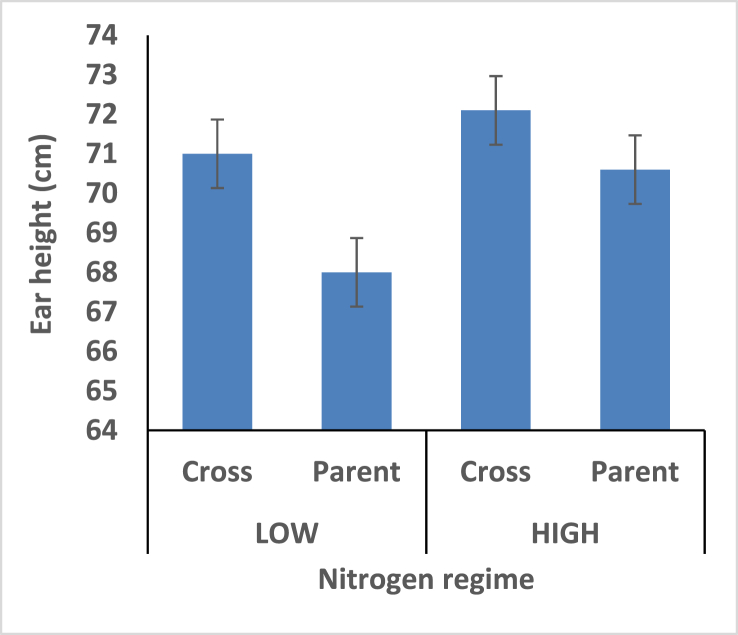
Effect of interaction between nitrogen fertilization and maize hybrids, open pollinated variety on Ear height.

**Figure 6:**
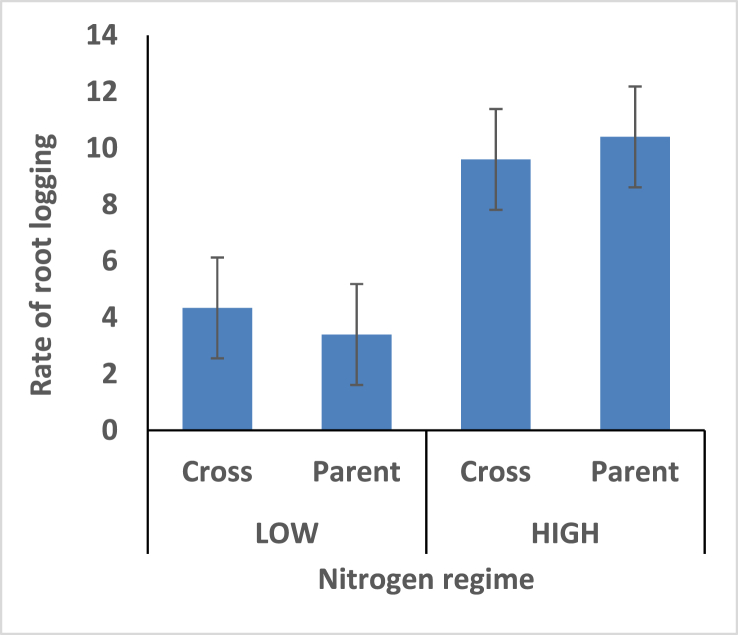
Effect of interaction between nitrogen fertilization and maize hybrids, open pollinated variety on Root lodging.

**Figure 7:**
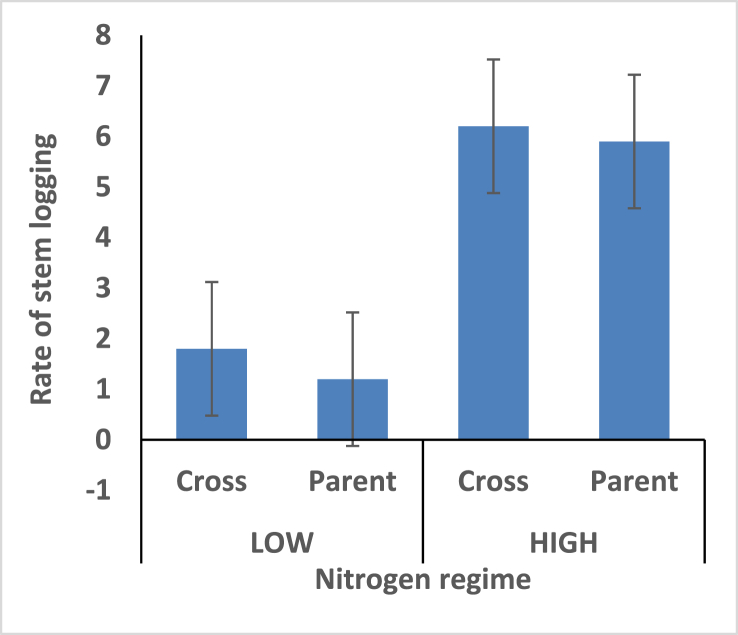
Effect of interaction between nitrogen fertilization and maize hybrids, open pollinated variety on stem lodging.

**Figure 8:**
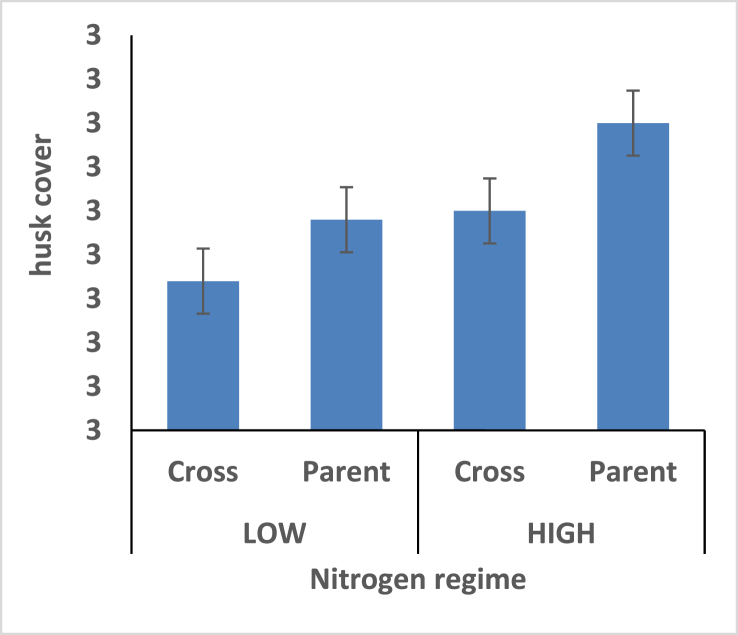
Effect of interaction between nitrogen fertilization and maize hybrids, open pollinated variety on husk cover.

**Figure 9:**
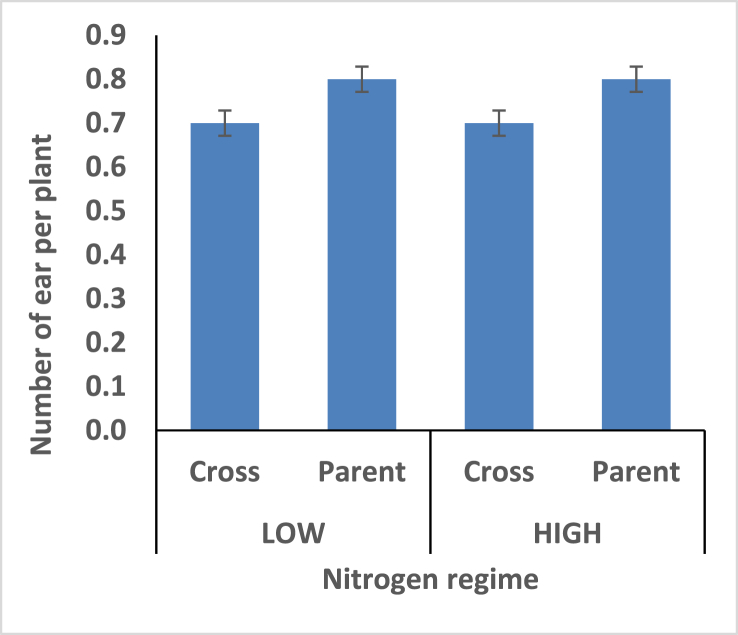
Effect of interaction between nitrogen fertilization and maize hybrids, open pollinated variety on Ear per plant.

Hybrids recorded shorter plant height under the two different nitrogen fertilizer conditions compare with open pollinated parents (Figure 4). Thus the hybrids is better for mechanical harvesting. Taller ear height were observed among the hybrids compare with the open pollinated parents; rate of stem and root lodging were consequently higher among the hybrids (Figures 5, 6, and 7). Hybrid had better husk cover under both nitrogen fertilizer regime (Figure 8); that's they will be less susceptible to Insect and Animal attack. Mean difference of ear per plant between the maize hybrids and open pollinated parents was not significant (Figure 9).

According to Xin Li 2015 [25], genes of plants produced from the same parent variety – sometimes cancel each other out. Combining the right varieties means those genes are no longer canceling each other out, unleashing the potential for desirable traits to manifest in the hybrid. He also said multiple genes govern a plant's total height. For instance, some genes may only influence the base of the plant, while other genes affect the entire plant. Untangling all those connections also points to how hybrids may outperform both parent varieties.

## 5 Conclusion

Parent versus hybrid effects were significant for grain yield, days to 50% anthesis, stalk lodging, plant and ear aspects. Days to flowering appeared to be earlier among the parental populations compared to the hybrids under high N environments. Low-N environment seemed to favor shorter day to silk for maize hybrid, however, high nitrogen soil condition appeared to increase day to silk It is an evidence that flowering and consequently maturity time is delayed for hybrid under favourable nitrogen soil environments.

Grain yield ranged from 1.6 t/ha^−1^ to 2.98 t/ha^−1^ for hybrids and 1.2 t/ha^−1^ to 2.2 t/ha^−1^ for parents in high-N environments. Higher grain yields were recorded among the hybrids compared to the parents under low-N condition is an evidence of tolerance of hybrid to low nitrogen environment compare to parental populations for the production of high grain yield.

Furthermore, that Era 1 versus era 2 and (Era 1 versus era 2) x environment mean square components were not significant for all the traits measured under low and high-N, suggests that there were no substantial differences between the means of the genotypes in the two eras and that their performances remained stable across the two locations.

## Declarations

### Author contribution statement

Sunday Ayodele Ige: Conceived and designed the experiments; Performed the experiments; Contributed reagents, materials, analysis tools or data; Wrote the paper.

Omolaran Bello: Performed the experiments.

Stephen Abolusoro: Performed the experiments; Analyzed and interpreted the data.

Charity Aremu: Performed the experiments; Contributed reagents, materials, analysis tools or data.

### Funding statement

This research did not receive any specific grant from funding agencies in the public, commercial, or not-for-profit sectors.

### Data availability statement

Data will be made available on request.

### Declaration of interests statement

The authors declare no conflict of interest.

### Additional information

No additional information is available for this paper.
